# Childhood exposure to armed conflict and nutritional health outcomes in Nigeria

**DOI:** 10.1186/s13031-023-00513-0

**Published:** 2023-03-29

**Authors:** Olusesan Ayodeji Makinde, Emmanuel Olamijuwon, Ifeanyi Mgbachi, Ryoko Sato

**Affiliations:** 1 Viable Knowledge Masters, Plot C114, First Avenue, Gwarimpa, Abuja, Federal Capital Territory Nigeria; 2 Viable Helpers Development Organization, Abuja, Federal Capital Territory Nigeria; 3 School of Geography and Sustainable Development, University of St Andrews, St Andrews, KY16 9AL UK; 4 Harvard University, 677 Huntington Ave, Boston, MA 02115 USA

**Keywords:** Armed conflict, Nigeria, Malnutrition, Vulnerable populations

## Abstract

**Background:**

Armed conflicts are associated with an increased risk of food insecurity, the leading cause of malnutrition in low-and-middle-income countries. Multiple studies have uncovered significant influences of childhood malnutrition on children’s overall health and development. As a result, it is increasingly important to understand how childhood experience of armed conflict intersects with childhood malnutrition in conflict-prone countries like Nigeria. This study examined the association between different measures of childhood experiences of armed conflicts and the nutritional health outcomes of children aged 36–59 months.

**Methods:**

We used data from the Nigeria Demographic and Health Survey linked with Uppsala Conflict Data Program Geo-Referenced Events Dataset using geographic identifiers. Multilevel regression models were fitted on a sample of 4226 children aged 36–59 months.

**Results:**

The prevalence of stunting, underweight and wasting was 35%, 20% and 3%, respectively. Armed conflicts were mostly recorded in the North-eastern states of Borno (222 episodes) and Adamawa (24 episodes). Exposure to armed conflicts ranged from 0 (no experience of armed conflict) to 3.75 conflicts per month since the child’s birth. An increase in the frequency of armed conflicts is associated with increased odds of childhood stunting [AOR = 2.52, 95%CI: 1.96–3.25] and underweight [AOR = 2.33, 95%CI: 1.19–4.59] but not wasting. The intensity of armed conflict was only marginally associated with stunting and underweight but not wasting. Longer conflicts that occurred in the last year were also associated with the odds of stunting [AOR = 1.25, 95%CI: 1.17–1.33] and underweight [AOR = 1.19, 95%CI: 1.11–1.26] but not wasting.

**Conclusion:**

Childhood exposure to armed conflict is associated with long-term malnutrition in children aged 36–59 months in Nigeria. Strategies that aim to end childhood malnutrition could target children exposed to armed conflicts.

**Supplementary Information:**

The online version contains supplementary material available at 10.1186/s13031-023-00513-0.

## Introduction

Armed conflicts are associated with an increased risk of food insecurity [1, 2], the leading cause of malnutrition in low-and-middle-income countries [3–6]. Food insecurity occurs when there is physical inaccessibility to food or the lack of social and economic access to food [7]. More than 112 million malnourished children live in areas exposed to conflict, and this number is equivalent to about two-thirds of all malnourished children in low-and-middle-income countries [1]. The number of people requiring food assistance due to conflicts and natural disasters has continued to rise. It is estimated that more than 40 million people will be facing food shortages, requiring food aid as a result of these causes by September 2020 [8].

Malnutrition is a major contributor to childhood morbidity and mortality, contributing not just in terms of its immediate influence on children’s health but also its long-term influence on cognitive development, individual and national economic growth and premature death [9–12]. According to the World Health Organization, ‘malnutrition refers to deficiencies, excesses or imbalances in a person’s intake of energy and/or nutrients’ [13]. There are two forms of malnutrition: undernutrition and over-nutrition [13]. Undernutrition which is our focus in this study, is of three types: micronutrient undernutrition, secondary undernutrition and protein-energy undernutrition [13]. It is estimated that 1 in 4 under-five children are stunted, and more than 49 million are wasted globally, with the majority of them living in sub-Saharan Africa [14]. According to the 2018 Nigeria Demographic and Health Survey, 37% of children aged 6–59 months were stunted, 7% were wasted, and 22% were underweight [15].

About 53% of all child deaths can be attributed to being underweight [16]. An analysis of 10 longitudinal surveys revealed that being underweight conferred an increased risk of death from infectious diseases. The fraction of diseases attributable to being underweight was 61% for diarrhoea, 57% for malaria, 53% for pneumonia, 45% for measles, and 53% for other infectious diseases [16]. Nigeria has the highest number of stunted and wasted children in sub-Saharan Africa, with the Northeast and Northwest regions of the country bearing this burden disproportionately [17, 18].

Malnutrition is caused by a wide range of factors, with poverty being the leader. Other causes include an inadequate and poor diet, inadequate household food security, poor health services, unhealthy environment and poor maternal/caregiver child care services [19, 20]. In conflict situations, these factors become even more pronounced and aggravated, causing a worsening of the nutritional status of children and other vulnerable groups [20–22].

### Conflict and child nutritional status

**Fig. 1:**
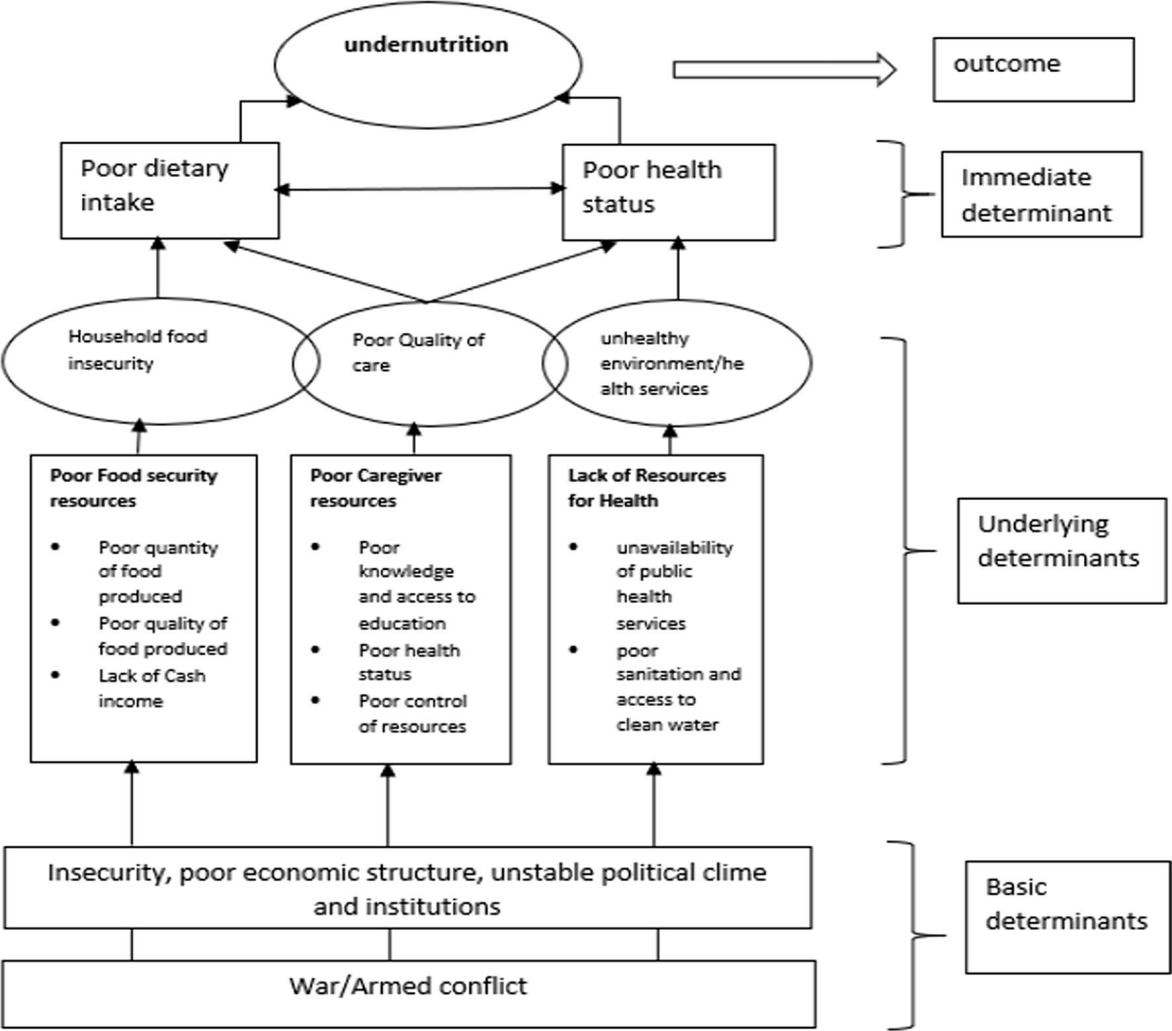
Conceptual Framework on Malnutrition–Adapted from UNICEF

Conflicts can exacerbate malnutrition in multiple ways. The malnutrition conceptual framework by UNICEF, as presented in Fig. 1, provides an explanatory framework that links conflict to undernutrition in children under five years. Undernutrition and death among children and other vulnerable groups exposed to armed conflicts can often be attributed to poor dietary intake and poor health status [23]. At the household level, malnutrition is driven by food insecurity, unhealthy environment, lack of access to health services and poor maternal and infant care. Accessibility is an important factor that must be considered in order to ensure adequate nutritional status for children. Conflict hinders accessibility–access to a stable environment to grow crops, access to sufficient food supplies, access to safe drinking water and access to health care facilities and personnel [19].

According to UNDP, more than 80% of war victims are women and children [24]. In conflict situations, children are either killed, disabled, orphaned, made homeless or separated from their parents or caregivers [25]. Widespread malnutrition and mortality among vulnerable women and children are significant features of many conflict situations [26–28]. This was seen in the Ethiopia-Eritrea war in 1999–2000 and in Burundi, where children experienced higher levels of stunting than children not affected by the conflicts [29]. As the conflict persists, the scale of malnutrition usually rises [27, 30]. Children that live in areas of armed insurgency are at higher risk of growth faltering [31, 32], developmental delay [33], wasting [34, 35] and being underweight [21, 36]. As a result of the interrelationship between conflict and malnutrition, nutrition interventions are significant components of humanitarian response in conflict situations.

Information on the nutritional status of children affected by armed conflict in Nigeria is limited. Estimates suggest that about 40% of the children exposed to the Nigeria-Biafra civil war in 1973 were malnourished [31]. Other studies have looked at the nutritional status of children in Internally Displaced Persons (IDP) camps [22, 32–35], both as a result of conflict and natural disasters. Dunn investigated the impact of the Boko Haram insurgency on childhood wasting [36], while Tyndall and colleagues examined the relationship between armed conflict and reproductive, maternal, newborn and child health and nutrition status and services in the Northeast region of Nigeria [37].

In Nigeria, the ongoing Boko Haram insurgency in the Northeast, which began in 2009, has affected healthcare facilities, health workers, schools and pupils, security outfits, and civilians. About 1.7 million people have been displaced in Borno, Adamawa, and Yobe states [38]. Of those displaced, more than 50% are children, including many unaccompanied or crisis-orphaned [39, 40]. Other forms of violent conflicts, such as farmer-herder clashes [41] and banditry [42], have also emerged across the country, leading to the displacement of residents and negatively affecting population health. It’s worth noting that the problem of undernutrition in children under-five in the Northeast and Northwest regions predates the Boko Haram insurgency. Estimates from the 2003 and 2008 Nigeria Demographic and Health Surveys (NDHS) suggest that more children in the Northwest geopolitical zone (out of the five zones remaining) were stunted and underweight, while the Northeast region had the highest percentage of children that were wasted [43]. However, the scale and intensity of armed conflicts could further exacerbate health outcomes for those who are already socioeconomically disadvantaged by shrinking the level of involvement of state actors in healthcare service delivery [44]. For example, the re-emergence of the Wild Polio Virus in 2016 was attributed to the insurgency, which prevented vaccinators from reaching some areas [45].

The emerging nature of various conflicts across the country necessitates investigation to determine the association of these conflicts with the nutritional status of children, who are most vulnerable in these conflicts. Therefore, this study leverages a nationally representative dataset of children in all states and regions of the country to elucidate the association between different dimensions of childhood experiences of armed conflicts and the nutritional health outcomes of children in Nigeria.

## Methods

### Data

The data for this study were obtained primarily from two sources. Individual-level data on demographics and nutritional health were obtained from the children’s module of the most recent demographic and health survey conducted in Nigeria (NDHS) in 2018. The NDHS is a large-scale national survey encompassing demographic and health information on men, women, children, and households. The dataset uses population sampling frames making the datasets representative at different levels, i.e. national, regional and household levels [46]. Furthermore, the DHS program routinely collects geographic information in all surveyed countries. The geographic data includes information on the geolocation of each sampled household in the dataset. To enhance the confidentiality of the respondents, the DHS randomly displaced/offset the GPS latitude/longitude positions for urban clusters to about two kilometres and 5 kms for rural clusters. About 1% of the rural clusters are also displaced up to 10 kms. These random displacements are restricted so that the geographic locations remain within the country and the survey region. The displacement for the 2018 NDHS data was particularly restricted to the local government areas (the country’s lowest administrative level). Overall, the survey’s rich information on maternal and child health and the availability of geographic information make it a valuable resource for studying the association between the environment and child nutritional health outcomes.

Information about children’s experience of armed conflict was obtained from the Uppsala Conflict Data Program (UCDP) Geo-referenced Event Dataset (UCDP GED). This dataset has detailed information on armed conflict events between 1989 and 2017 globally [47], including the ones in Nigeria. In addition, the data contains information on the timing of conflicts, GPS coordinates of conflict locations, estimates of total fatalities from each conflict, and a link to news articles reporting the event. Furthermore, the dataset includes information about whether the conflicts are state-sponsored, non-state sponsored, as well as specific groups involved or affected.

### Study sample

The NDHS dataset comprised 33,924 children under five years drawn from the six geopolitical zones in Nigeria. In our analysis, we focus only on children whose anthropometric data were collected. We also excluded children younger than 36 months because anthropometric data indicating absolute deprivation has been argued to be less reliable in very early childhood [48–50]. As a result, our final analytical sample for the study comprised 4299 children aged 36–59 months whose anthropometric data were collected.

### Measures

#### Child nutritional health outcomes

We assessed different forms of children’s nutritional health status that encompasses underweight, stunting, and wasting. These outcomes were assessed using anthropometric data on children’s weight, height and age at the time of data collection. Children’s height was measured using a measuring board to the nearest 0.1 cm, while children’s weight was measured using a pediatric scale also to the nearest 0.1 kg. To minimise measurement errors that may arise randomly or systematically, two measurements were taken for each child’s height and weight to ensure agreement within 0.1 cm and 0.1 kg, respectively. Using the information retrieved from height and weight, the DHS then codes children according to the number of standard deviations they are below/above the World Health Organization’s standard for healthy growth.

Using these data, we created three distinct binary dependent variables as an indication of whether the children are stunted, wasted or underweight. We grouped children as being *stunted* (1) if their height for age score was less than -2 Z score or standard deviation of the reference population of WHO Multicentre Growth Study [51, 52] or nutritionally healthy (0) if they were not stunted and not wasted and not underweight. Children were also classified as being *wasted* (1) if the weight for height score was less than -2 Z score or standard deviation of the reference population of WHO Multicentre Growth Study [51, 52] or nutritionally healthy (0) if they were not stunted and not wasted and not underweight. Similarly, we grouped children as being *underweight* if their weight for age score was less than -2 Z score or standard deviation of the reference population of WHO Multicentre Growth Study [51, 52] or nutritionally healthy (0) if they were not stunted and not wasted and not underweight.

#### Experience of armed conflict

Children’s experience of armed conflict was assessed based on the occurrence of conflicts with more than **25** fatalities that occurred within an estimated 10 km radius of the cluster (not accounting for displacement error) in relation to the child’s household of residence [47]. We created three different measures of armed conflict, including the frequency of occurrence of armed conflict, the intensity of the armed conflicts and the duration of armed conflicts. We merged the geo-coordinates of each child in the NDHS data with the geolocations of armed conflicts recorded in the UCDP geo-referenced event dataset using the **fuzzyjoin **package in R [53]. Using the information on the date of the interview, date of armed conflict and the date of birth for each child, we retained only armed conflicts that happened between the period that each child was born and the time of the interview.

Furthermore, all the measures of armed conflict were normalised by dividing the aggregate counts of conflicts experienced by the age of the child to control for the effect of age (older children experiencing more armed conflicts). As a result, the measures correspond to the monthly average since the child’s birth. The *frequency of occurrence of armed conflict* was assessed based on the aggregated counts of all armed conflicts experienced by each child since birth. Values for the variable ranged from 0 *(no experience of armed conflict)* to 3.75 *(an average of about four armed conflicts per month since the birth of the child)*. The *duration of armed conflicts* assessed the duration of individual conflicts that the children experienced as well as the cumulative number of days per month. Values for the variable ranged from 0 *(no day per month)* to 6.67 *(an average of 7 days per month for the conflicts experienced by the child since birth)*. Lastly, the *intensity of conflicts* was assessed based on the aggregated counts of deaths from all armed conflicts that each child experienced since birth. The values for this measure ranged from 0 *(no casualty reported for any armed conflict)* to 47.75 *(an average of 48 casualties from all armed conflicts experienced by the child per month since the birth of the child)*.

#### Covariates

Our analysis included other maternal, household, and child characteristics as covariates. These include maternal age, maternal highest educational attainment, maternal age group, maternal BMI, household wealth, place of residence, child’s age, child’s anaemia status, and an indication of whether the child was breastfed, breastfeeding or never breastfed.

### Analysis strategy

In the first step of our analysis, weighted frequency distributions were obtained to describe the demographic profile of the children as well as the levels of fatality from armed conflicts and childhood malnutrition across the states (first geographic administrative level). Associations between children’s exposure to armed conflict and nutritional status were assessed using multiple multilevel multivariable binary logistic regression models. Due to collinearity, we specified each of the main predictor variables with the outcomes in separate models while controlling for other relevant characteristics such as maternal age, maternal highest educational attainment, maternal age group, maternal BMI, household wealth, place of residence, child’s age, child’s anaemia status, and an indication of whether the child was breastfed, actively breastfeeding, or never breastfed. In a secondary analysis, we restricted all the measures of armed conflict to those experienced within the last year preceding the survey to delineate the association between a recent experience of armed conflicts and children’s nutritional health. All data wrangling and visualisation were done in R, while STATA was used for fitting the multilevel multivariable binary logistic regression models. The analyses were weighted to account for the complex design of the sampling.

## Results

### Descriptive profile of children in the sample

Table 1 shows descriptive characteristics of the children, the demographics of their mothers, as well as the household in which they reside. About 51% of the children are girls, and about 51% are aged three years. Less than 2% of the children were never breastfed, and more than half had severe (2%), moderate (32%), or mild (26%) anaemia. More than half of the children’s mothers had less than secondary education, and the majority of the mothers had a healthy body mass. The majority of the children reside in rural areas, and nearly 40% reside in the ‘richer or richest households.

**Table 1:** Sociodemographic, health, and maternal characteristics of children aged 36–59 months

Maternal and household characteristics	*n*	%	Child characteristics and residence	*n*	%
*Maternal education*			*Child’s age*		
No education	1668	40.44	3 years	2122	50.80
Primary	773	16.87	4 years	2104	49.21
Secondary	1416	33.53	*Childhood anaemia*		
Higher	369	9.15	Severe	106	2.26
*Maternal BMI*			Moderate	1397	31.90
< 18.5	359	8.45	Mild	1076	25.64
18.5–24.9	2640	61.46	Not anaemic	1647	40.20
25.0–29.9	813	19.77	*Child ever breastfed*		
30 +	414	10.32	Previously Breastfed	4117	97.35
*Household wealth*			Never Breastfed	80	1.98
Poorest	867	19.19	Still Breastfeeding	29	0.67
Poorer	830	19.43	*Sex of child*		
Middle	937	20.88	Male	2104	50.71
Richer	880	20.56	Female	2122	49.21
Richest	712	19.94	*Children’s nutritional health status*^*†*^		
*Place of residence*			Stunted	1481	35.43
Urban	1631	43.53	Wasted	125	2.91
Rural	2595	56.47	Underweight	849	20.11
Total	4226	100.0%	Healthy	2598	61.09

Frequency distributions are unweighted, while percentage distributions (%) are weighted to adjust for the complex design of the survey; ^*†*﻿^-The sub-categories are not mutually exclusive; thus, n (and %) exceeds the total sample size (and 100%)

### The magnitude of childhood malnutrition and children’s experience of armed conflict

**Fig. 2:**
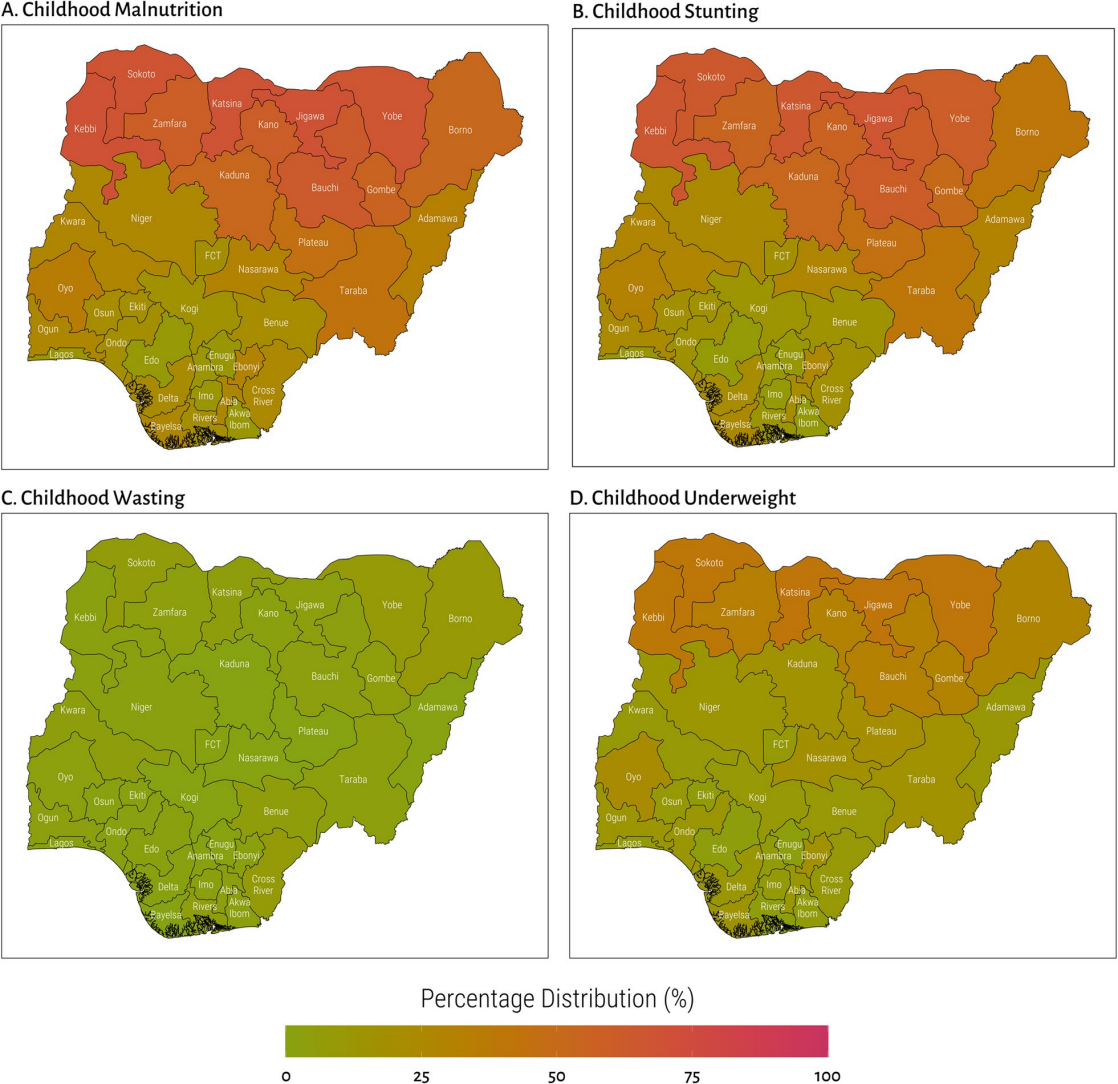
Distribution of childhood malnutrition among children aged 36–59 months across the 36 states and federal capital territory

About a third of the children were malnourished, with a higher percentage of children either stunted (35%) or underweight (20%) than being wasted (3%). Figure 2 presents the distribution of childhood malnutrition across the 36 states and the Federal capital territory. As presented in Panel A of the figure, the level of malnutrition is higher in the northern states compared to the states in the southern region. Precisely, more than 65% of the children in Katsina (69%), Kebbi (69%), Jigawa (69%), Sokoto (67%), and Yobe (67%) states were wasted, stunted, and or underweight compared to about 10% of children in Edo (9%), Akwa Ibom (11%), and Enugu (11%) states (see Additional file 1: Table S1). The pattern of childhood stunting (panel B) and underweight (panel D) are also consistent with the overall malnutrition. A larger percentage of children in the north-west region are stunted or underweight compared to the children in the southern region. The overall prevalence of wasting was low across the states. Only about 16% of children in Borno state, 12% in Katsina and 11% in Yobe were wasted compared to almost none of the children in several other states.

**Fig. 3:**
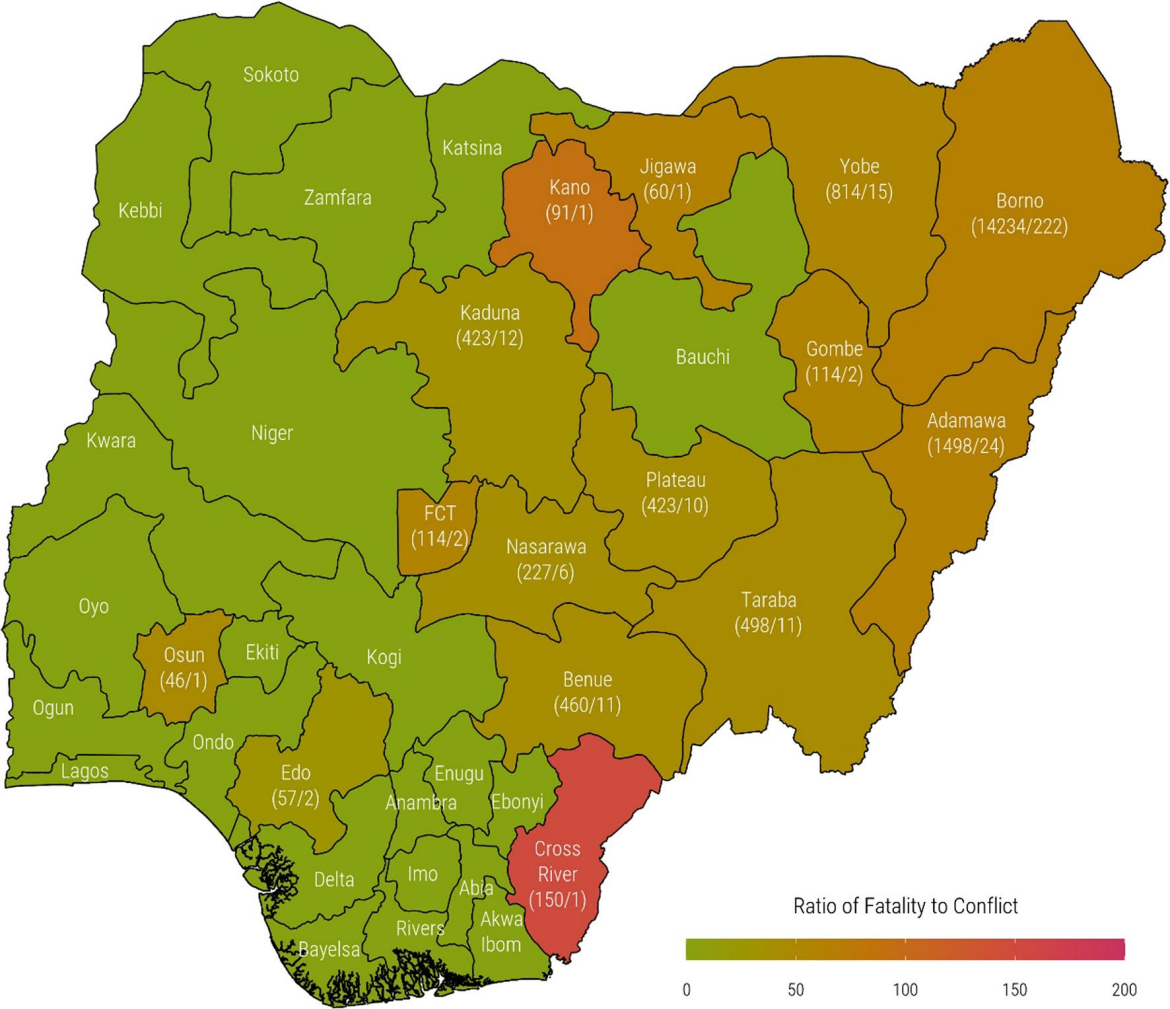
Ratio of fatalities to armed conflicts across the 36 states and Federal Capital Territory in Nigeria between 2013 and 2018

Figure 3 presents the distribution of armed conflicts, fatalities resulting from the conflicts and the ratio of armed conflicts to fatalities that occurred in the states between 2013 and 2018. As presented in the figure, the majority of states in the Northern region of the country experienced a significant number of armed conflicts. Precisely, Borno state experienced 222 armed conflicts during the period that resulted in a total of 14,234 fatalities. Adamawa state also experienced 24 conflicts that resulted in the death of 1498 people. There were also a few occurrences of armed conflicts in some of the Southern States. Osun state experienced one armed conflict that led to the death of 46 people, while Cross River state experienced one armed conflict that resulted in 150 fatalities. The intensity of the armed conflicts appears to be higher in Kano and Cross River States.

### Associations between children’s experience of armed conflict and childhood malnutrition

Table 2 presents the results of a multivariate logistic regression model showing associations between the different measures of armed conflict and children’s nutritional health. The full model results are available in Additional file 1: Tables S2 and S3. The results showed that exposure to a higher number of armed conflict per month during childhood [Adjusted odd ratios (AOR) = 2.52, 95%CI = 1.96–3.25] was associated with a higher likelihood of stunting among the children. Similarly, exposure to a higher number of fatalities from armed conflict was associated with a higher likelihood of childhood stunting. The result also showed a positive association between childhood underweight and the frequency of armed conflicts [AOR: 2.33, 95%CI: 1.19–4.59] as well as between the intensity of armed conflict (albeit weak) and underweight [AOR = 1.01, 95%CI: 1.00–1.03]. We also examined the association between recent children’s experience of armed conflict and each of the nutritional health outcomes (Table 3). The results presented in Table 3 show that a higher number of recent (within the last one year) childhood experiences of armed conflict [AOR = 1.22, 95%CI = 1.11–1.34], the intensity of armed conflict and the duration of the armed conflict [AOR = 1.25, 95%CI = 1.17–1.33] were associated with a higher likelihood of stunting among the children. The duration of recent armed conflicts within the last one year [AOR = 1.19, 95%CI = 1.11–1.26] was also associated with children being underweight.

**Table 2:** Association between childhood (any) experience of armed conflict and nutritional health status of children aged 36–59 months in Nigeria

Measures of childhood experiences of armed conflicts	Adjusted odd ratios [95% Confidence Intervals]		
	Stunted	Wasting	Underweight
Frequency of attack	2.52*** [1.96,3.25]	0.30 [0.04,1.98]	2.33* [1.19,4.59]
Intensity of attack	1.01*** [1.01,1.01]	0.97 [0.93,1.00]	1.01** [1.00,1.03]
Duration of attack	1.21 [0.84,1.75]	0.82 [0.02,35.27]	1.02 [0.59,1.74]

Models adjusted for maternal age, maternal highest educational attainment, maternal age group, maternal BMI, household wealth, place of residence, child’s age, child’s anaemia status, breastfeeding. See Additional file 1: Table S2 for full model results

***p < 0.001, **p < 0.01, *p < 0.05

**Table 3:** Association between childhood recent (last one year) experience of armed conflict and nutritional health status of children aged 36–59 months in Nigeria

Measures of childhood experiences of armed conflicts	Adjusted odds ratios [95% Confidence Intervals]		
	Stunted	Wasting	Underweight
Frequency of attack	1.22*** [1.11,1.34]	0.86 [0.42,1.77]	1.1 [0.94,1.29]
Intensity of attack	1.00* [1.00,1.01]	0.99 [0.97,1.01]	1.00 [1.00,1.00]
Duration of attack	1.25*** [1.17,1.33]	0.85 [0.45,1.60]	1.19*** [1.11,1.26]

Models adjusted for maternal age, maternal highest educational attainment, maternal age group, maternal BMI, household wealth, place of residence, child’s age, child’s anaemia status, breastfeeding. See Additional file 1: Table S3 for full model results

***p < 0.001, **p < 0.01, *p < 0.05

## Discussion

Conflicts have long been linked to the emergence and severity of undernutrition of various forms across the world. The ongoing insurgency in the Northeast is noted to have resulted in the destabilisation of food supplies and caused various social inadequacies in the process. Despite the potential importance and consequence of conflicts on nutritional outcomes among children in Nigeria, the evidence has been scarce. In general, both the level of malnutrition and the frequency of armed conflicts were higher in the northern states compared to the states in the southern region. This paper employed a rigorous statistical methodology to illuminate the association between children’s experience of armed conflict and nutritional outcomes in children using a global database of armed conflict data as well as the 2018 Nigeria Demographic and Health Survey.

Our analysis revealed a high level of childhood malnutrition, mostly in states in the Northeast and Northwest regions. This finding is consistent with several other previous studies and mirrors the pattern of socioeconomic disadvantage in other studies. We also observed that the frequency and intensity of armed conflict were concentrated in the Northeast region, with a few episodes in the Southern region. This finding highlights that children in Nigeria are not only affected by conflicts related to terrorism but also due to ethnic and communal clashes, as identified in Cross River and Osun states. Surprisingly, our analysis uncovers a high level of childhood malnutrition in a few states in the Northwest region which are not severely affected by armed conflict. However, these states–Sokoto, Jigawa, and Kebbi states suffer from other social disadvantages and are ranked poorest on the Multidimensional Poverty Index. As a result, it’s likely that armed conflicts are associated with childhood malnutrition in some states, while in others, it may be due to intersecting disadvantages such as poverty and conflict.

Furthermore, we found evidence in support of the conceptual premise that children’s healthy nutritional status was negatively associated with their experience of armed conflicts. In particular, the more frequent and intense the conflicts are, the more children are likely to be stunted and underweight. On the other hand, children’s experience of armed conflicts was not associated with children’s short-term nutritional status. The duration of the individual conflicts also was not significantly associated with nutritional status among Nigerian children. These main findings hold even after focusing on the conflicts that occurred recently, within one year from the survey period: the high frequency and intensity of conflicts were negatively associated with long-term nutritional outcomes, but they did not influence the short-term outcome. In addition to these results, we also found that the duration of recent conflicts was negatively associated with long-term nutritional outcomes.

Our finding that the frequency and duration of armed conflict are associated with long-term nutritional health outcomes is consistent with those observed in previous studies [21, 54–57]. According to Institute for Security Studies (ISS) [38], household food insecurity results in three ways: firstly, the loss of family members reduces the likelihood that an infant receives adequate care and nutrition. Secondly, stopping economic activities causes food insecurity and limits families’ ability to care for their children. Finally, conflicts might destroy farmlands and other means of livelihood [38]. The conflict in Somalia provides a good example of this scenario, where over 3 million people were displaced upon resumption of fighting in 2006. This led to disruption in access to food and basic amenities, causing many people to be displaced internally and to neighbouring countries. In IDP camps and feeding stations, there have been reports of crowding, with the attendant shortage of human disposal systems and the lack of treated water supplies, as well as corruption and pilfering of foodstuff meant for displaced persons, contributing to diseases and subsequent malnutrition [58].

Consequently, we observed no association between armed conflict and a short-term nutritional outcome like wasting. This finding is in contrast with those established in previous studies. For example, the Boko Haram insurgency in North-East Nigeria has been linked with an increase in the prevalence of wasting in children [36]. However, there is also evidence of a coordinated response by the government and its development partners to mitigate the consequences of the conflict and displacement on children in various affected communities in Northeast Nigeria [37, 59–61]. For example, Tyndall et al. [37] observed an improvement in measles vaccination coverage following the arrival of international humanitarian aid in an armed conflict-affected state in 2016. The World Food Programme has also led other international development partners and the government to provide food aid distribution to those affected by the conflict [62]. The Doctors without Borders reported that in one month, it distributed more than 810 tonnes of food to people in Maiduguri, food enough for each family for a couple of weeks [63]. These food aids could have alleviated the immediate nutritional needs and outcomes seen in children in the many of the affected communities.

This study is not without limitations. First, we used a large-scale, nationally representative survey of children in selected households across Nigeria. As with other cross-sectional data analyses, our results should be interpreted with caution. Our goal was to identify associations between various dimensions of childhood experience of armed conflict and children’s nutritional health and not to identify causal effects because the temporal sequence of the events could not be ascertained. In addition, our models incorporated the current characteristics of children, their parents, and their households at the time of the survey. However, families may have moved and changed their place of residence since the time that the children were born and thus may not have experienced all the armed conflict associated with a geographic location or experienced more in their previous place of residence. Estimates from our assessments suggest that mobility patterns, particularly in our sample, were very low in the northern regions compared to the southern region. As a result, we do not expect that mobility patterns would significantly affect the magnitude and direction of association identified in this study.

Secondly, the displacement of the geographic position of households in the DHS is likely to introduce a misclassification bias. However, given that the displacement is within two and five kilometres for urban and rural clusters, respectively, we anticipate that the impact of these displacements on our estimates and the hypothesised relationship would be minimal. Our analytic sample comprised only a subset of the DHS sample. That is, children with complete anthropometric data and children that were alive and at least 36 months old at the time of data collection. It is known that malnutrition increases the risk of morbidity and mortality in children, and armed conflict could also exacerbate mortality risks in children under five. As a result of this survivorship bias, the findings may not be inferred beyond our study population. Furthermore, our analysis adjusts for several important socioeconomic characteristics, household characteristics, and child-related factors. However, we could also not adjust for several other relevant characteristics, such as birth weight which is known to influence stunting [64], and maternal experience of spousal violence primarily due to a high level of missing values on these measures. For example, about 75% of children in our sample were not weighted at birth, and the DHS only collects data on spousal violence from a random sample of women.

Lastly, we adapted an established definition of armed conflicts and classified children as having experienced armed conflicts if there has been any violence resulting in at least 25 mortalities in their community or another close community. However, using a cut-off of 25 could introduce a misclassification bias, particularly since frequent low-fatality conflicts can also cause significant disruptions with negative impacts on children’s health. Despite these limitations, our study contributes to the literature in several important ways. Our analysis incorporates all forms of armed conflicts, including those related to terrorism, ethnic or communal clashes, religious intolerance, and political conflicts. We also examined different dimensions of armed conflict, encompassing frequency, duration, and intensity.

## Conclusion

We found that armed conflicts in Nigeria are associated with long-term nutritional outcomes for children. This has great implications for the health, future cognitive performance, and survival of the children. Several states in the northern region of the country, which bore a greater burden of malnutrition, are also worse affected by the armed conflicts, with sporadic occurrences of armed conflicts in the south. There is a need for interventions to address the rising insecurity and armed conflicts in Nigeria. To address the malnutrition burden attributable to armed conflicts, a holistic look at the issue of security, considering food security and other social and economic factors that affect food availability in the home, is necessary. Additional studies are needed to further understand the level of contribution of the conflicts to undernutrition across the country.

## Supplementary Information

**Additional file 1. Table S1:** Percentage distribution of indices of malnutrition by state. **Table S2:** Association between childhood lifetime and recent experience of armed conflict and childhood stunting of children aged 36-59 years in Nigeria. **Table S3:** Association between childhood lifetime and recent experience of armed conflict and childhood wasting of children aged 36-59 years in Nigeria. **Table S4:** Association between childhood lifetime and recent experience of armed conflict and childhood underweight of children aged 36-59 years in Nigeria.

## Data Availability

The data underlying the results presented in the study are publicly available from the DHS Program (https://dhsprogram.com/data) and the Uppsala Conflict Data Program (https://ucdp.uu.se/downloads/) websites. Interested researchers will be required to register on the website to access the dataset. We confirm that we do not have any special privileges that others would not have.
