# Supplementary material for: Childhood exposure to armed conflict and nutritional health outcomes in Nigeria
Source: Confl Health. 2023 Mar 29;17:15. doi: 10.1186/s13031-023-00513-0 (PMC10053485; doi:10.1186/s13031-023-00513-0)
Supplement: Supplementary file 1 — Additional file 1. Table S1: Percentage distribution of indices of malnutrition by state. Table S2: Association between childhood lifetime and recent experience of armed conflict and childhood stunting of children aged 36-59 years in Nigeria. Table S3: Association between childhood lifetime and recent experience of armed conflict and childhood wasting of children aged 36-59 years in Nigeria. Table S4: Association between childhood lifetime and recent experience of armed conflict and childhood underweight of children aged 36-59 years in Nigeria. [file 13031_2023_513_MOESM1_ESM.docx]

S1: Percentage distribution of indices of malnutrition by state

| **State** | **Stunted** | **Wasted** | **Underweight** | **Any Malnutrition** |  |
| --- | --- | --- | --- | --- | --- |
| Sokoto | 63.7 | 6.2 | 40.0 | 67.4 |  |
| Zamfara | 54.9 | 3.6 | 32.7 | 58.9 |  |
| Katsina | 64.7 | 4.8 | 41.6 | 68.8 |  |
| Jigawa | 67.2 | 3.4 | 41.4 | 69.2 |  |
| Yobe | 59.4 | 8.1 | 40.8 | 67.2 |  |
| Borno | 40.3 | 9.6 | 27.3 | 51.7 |  |
| Adamawa | 29.3 | 0.4 | 10.9 | 32.2 |  |
| Gombe | 49.9 | 4.4 | 29.4 | 55.3 |  |
| Bauchi | 60.3 | 2.8 | 31.2 | 62.7 |  |
| Kano | 56.5 | 2.3 | 30.7 | 59.3 |  |
| Kaduna | 52.5 | 0.0 | 16.4 | 52.5 |  |
| Kebbi | 65.7 | 1.6 | 38.7 | 69.4 |  |
| Niger | 23.9 | 1.7 | 12.7 | 25.0 |  |
| FCT | 17.9 | 0.6 | 8.1 | 18.5 |  |
| Nasarawa | 26.7 | 0.0 | 17.6 | 29.8 |  |
| Plateau | 45.6 | 0.0 | 19.3 | 45.6 |  |
| Taraba | 39.9 | 3.4 | 16.3 | 43.4 |  |
| Benue | 15.0 | 5.5 | 10.0 | 20.7 |  |
| Kogi | 13.4 | 0.0 | 9.0 | 16.4 |  |
| Kwara | 27.0 | 3.7 | 9.0 | 27.0 |  |
| Oyo | 30.8 | 2.6 | 22.4 | 35.5 |  |
| Osun | 17.6 | 0.9 | 8.9 | 21.1 |  |
| Ekiti | 16.6 | 1.0 | 6.7 | 19.2 |  |
| Ondo | 15.7 | 1.4 | 11.9 | 18.6 |  |
| Edo | 8.0 | 1.9 | 4.3 | 9.0 |  |
| Anambra | 10.1 | 0.8 | 6.5 | 12.7 |  |
| Enugu | 10.5 | 0.5 | 3.5 | 11.0 |  |
| Ebonyi | 25.4 | 2.0 | 16.2 | 29.4 |  |
| Cross River | 16.9 | 7.5 | 9.0 | 24.4 |  |
| Akwa Ibom | 8.0 | 2.8 | 6.6 | 10.9 |  |
| Abia | 20.6 | 2.8 | 11.6 | 25.6 |  |
| Imo | 8.4 | 2.0 | 7.7 | 12.0 |  |
| Rivers | 13.0 | 4.7 | 4.3 | 19.0 |  |
| Bayelsa | 23.9 | 0.0 | 13.8 | 26.4 |  |
| Delta | 20.5 | 1.1 | 11.8 | 22.2 |  |
| Lagos | 11.0 | 1.5 | 8.6 | 13.2 |  |
| Ogun | 23.1 | 3.1 | 12.6 | 26.0 |  |

*Note: FCT = Federal Capital Territory*

## S2: Association between childhood lifetime and recent experience of armed conflict and childhood stunting of children aged 36-59 years in Nigeria

|  | Lifetime experience of armed conflict and childhood stunting  Odd Ratios [95% Confidence Intervals] | | |  | Recent experience of armed conflict and childhood stunting  Odd Ratios [95% Confidence Intervals] | | | |
| --- | --- | --- | --- | --- | --- | --- | --- | --- |
| Frequency of attack | 2.52*** [1.96,3.25] |  |  |  | 1.22*** [1.11,1.34] |  |  |  |
| Intensity of attack |  | 1.01*** [1.01,1.01] |  |  |  | 1.00* [1.00,1.01] |  |  |
| Duration of attack |  |  | 1.21 [0.84,1.75] |  |  |  | 1.25*** [1.17,1.33] | |
| **Maternal Age Group** |  |  |  |  |  |  |  |  |
| 15-24 | Reference | Reference | Reference |  | Reference | Reference | Reference | |
| 25-34 | 0.93 [0.85,1.03] | 0.94 [0.85,1.03] | 0.94 [0.85,1.04] |  | 0.94 [0.85,1.04] | 0.94 [0.85,1.04] | 0.94 [0.85,1.04] | |
| 35+ | 0.91 [0.78,1.06] | 0.91 [0.78,1.06] | 0.91 [0.78,1.06] |  | 0.91 [0.78,1.06] | 0.91 [0.78,1.06] | 0.91 [0.78,1.06] | |
| **Maternal Education** |  |  |  |  |  |  |  |  |
| No education | Reference | Reference | Reference |  | Reference | Reference | Reference | |
| Primary | 0.94 [0.80,1.12] | 0.94 [0.79,1.12] | 0.94 [0.79,1.11] |  | 0.94 [0.79,1.12] | 0.94 [0.79,1.12] | 0.94 [0.80,1.11] | |
| Secondary | 0.64*** [0.54,0.76] | 0.64*** [0.54,0.76] | 0.64*** [0.54,0.75] |  | 0.64*** [0.54,0.76] | 0.64*** [0.54,0.75] | 0.64*** [0.54,0.75] | |
| Higher | 0.38*** [0.25,0.59] | 0.38*** [0.24,0.59] | 0.38*** [0.24,0.59] |  | 0.38*** [0.24,0.60] | 0.38*** [0.24,0.59] | 0.38*** [0.24,0.60] | |
| **Household Wealth** |  |  |  |  |  |  |  |  |
| Poorest | 1.08 [0.92,1.26] | 1.07 [0.92,1.25] | 1.07 [0.92,1.24] |  | 1.07 [0.92,1.26] | 1.07 [0.92,1.24] | 1.08 [0.92,1.26] | |
| Poorer | 1.09 [0.95,1.25] | 1.09 [0.95,1.25] | 1.08 [0.94,1.25] |  | 1.09 [0.95,1.25] | 1.09 [0.95,1.25] | 1.09 [0.95,1.25] | |
| Middle | Reference | Reference | Reference |  | Reference | Reference | Reference | |
| Richer | 0.68** [0.51,0.91] | 0.68** [0.51,0.91] | 0.69* [0.51,0.92] |  | 0.68** [0.51,0.90] | 0.68** [0.51,0.91] | 0.68** [0.51,0.90] | |
| Richest | 0.57*** [0.43,0.75] | 0.57*** [0.43,0.75] | 0.57*** [0.43,0.75] |  | 0.56*** [0.43,0.73] | 0.56*** [0.43,0.74] | 0.56*** [0.42,0.73] | |
| **Place of Residence** |  |  |  |  |  |  |  |  |
| Urban | Reference | Reference | Reference |  | Reference | Reference | Reference | |
| Rural | 1.16* [1.03,1.31] | 1.16* [1.03,1.30] | 1.14* [1.01,1.30] |  | 1.16* [1.03,1.30] | 1.14* [1.01,1.29] | 1.16* [1.03,1.30] | |
| **Maternal BMI** |  |  |  |  |  |  |  |  |
| < 18.5 | 0.98 [0.73,1.34] | 0.99 [0.73,1.34] | 0.99 [0.73,1.34] |  | 0.99 [0.73,1.34] | 0.99 [0.73,1.34] | 0.99 [0.73,1.34] | |
| 18.5-24.9 | Reference | Reference | Reference |  | Reference | Reference | Reference | |
| 25.0-29.9 | 0.82* [0.69,0.97] | 0.82* [0.69,0.97] | 0.82* [0.69,0.96] |  | 0.82* [0.69,0.97] | 0.82* [0.69,0.97] | 0.82* [0.69,0.97] | |
| 30+ | 0.47*** [0.34,0.67] | 0.48*** [0.34,0.67] | 0.48*** [0.34,0.68] |  | 0.48*** [0.34,0.67] | 0.48*** [0.34,0.68] | 0.47*** [0.33,0.67] | |
| **Child’s Sex** |  |  |  |  |  |  |  |  |
| Male | Reference | Reference | Reference |  | Reference | Reference | Reference | |
| Female | 0.95 [0.85,1.06] | 0.95 [0.85,1.06] | 0.95 [0.85,1.06] |  | 0.95 [0.85,1.07] | 0.95 [0.85,1.06] | 0.95 [0.85,1.07] | |
| **Child’s Age** |  |  |  |  |  |  |  |  |
| 3 Years | Reference | Reference | Reference |  | Reference | Reference | Reference | |
| 4 Years | 0.89* [0.80,0.98] | 0.89* [0.81,0.98] | 0.89* [0.81,0.98] |  | 0.89* [0.81,0.98] | 0.89* [0.81,0.98] | 0.89* [0.81,0.98] | |
| **Anaemia Levels** |  |  |  |  |  |  |  |  |
| Severe | 2.56*** [1.97,3.32] | 2.56*** [1.97,3.32] | 2.55*** [1.97,3.31] |  | 2.56*** [1.97,3.33] | 2.56*** [1.97,3.32] | 2.57*** [1.97,3.34] | |
| Moderate | 1.73*** [1.55,1.93] | 1.73*** [1.54,1.93] | 1.73*** [1.54,1.93] |  | 1.73*** [1.55,1.93] | 1.73*** [1.54,1.93] | 1.73*** [1.55,1.93] | |
| Mild | 1.23** [1.07,1.41] | 1.22** [1.07,1.41] | 1.22** [1.06,1.40] |  | 1.23** [1.07,1.41] | 1.23** [1.07,1.41] | 1.23** [1.07,1.41] | |
| Not anaemic | Reference | Reference | Reference |  | Reference | Reference | Reference | |
| **Breastfeeding** |  |  |  |  |  |  |  |  |
| Ever breastfed, not currently | Reference | Reference | Reference |  | Reference | Reference | Reference | |
| Never breastfed | 1.63*** [1.42,1.87] | 1.63*** [1.42,1.86] | 1.62*** [1.42,1.86] |  | 1.63*** [1.43,1.87] | 1.63*** [1.42,1.86] | 1.63*** [1.43,1.87] | |
| Still breastfeeding | 1.52 [0.59,3.89] | 1.52 [0.59,3.90] | 1.51 [0.58,3.90] |  | 1.5 [0.59,3.83] | 1.51 [0.58,3.88] | 1.5 [0.59,3.82] | |

## S3: Association between childhood lifetime and recent experience of armed conflict and childhood wasting of children aged 36-59 years in Nigeria

|  | Lifetime experience of armed conflict and childhood wasting  Odd Ratios [95% Confidence Intervals] | | |  | Recent experience of armed conflict and childhood wasting  Odd Ratios [95% Confidence Intervals] | | | |
| --- | --- | --- | --- | --- | --- | --- | --- | --- |
| Frequency of attack | 0.30 [0.04,1.98] |  |  |  | 0.86 [0.42,1.77] |  |  | |
| Intensity of attack |  | 0.97 [0.93,1.00] |  |  |  | 0.99 [0.97,1.01] |  | |
| Duration of attack |  |  | 0.82 [0.02,35.27] |  |  |  | 0.85 [0.45,1.60] | |
| **Maternal Age Group** |  |  |  |  |  |  |  |  |
| 15-24 | Reference | Reference | Reference |  | Reference | Reference | Reference | |
| 25-34 | 0.80 [0.50,1.27] | 0.80 [0.50,1.27] | 0.80 [0.50,1.26] |  | 0.80 [0.50,1.26] | 0.80 [0.50,1.26] | 0.80 [0.50,1.26] | |
| 35+ | 0.70 [0.34,1.45] | 0.70 [0.34,1.45] | 0.70 [0.34,1.46] |  | 0.70 [0.34,1.45] | 0.70 [0.34,1.45] | 0.70 [0.34,1.45] | |
| **Maternal Education** |  |  |  |  |  |  |  |  |
| No education | Reference | Reference | Reference |  | Reference | Reference | Reference | |
| Primary | 0.43* [0.20,0.96] | 0.43* [0.19,0.96] | 0.44* [0.20,0.95] |  | 0.43* [0.20,0.94] | 0.43* [0.20,0.94] | 0.43* [0.20,0.94] | |
| Secondary | 0.72 [0.48,1.10] | 0.72 [0.47,1.09] | 0.73 [0.50,1.07] |  | 0.73 [0.49,1.07] | 0.72 [0.49,1.07] | 0.73 [0.49,1.08] | |
| Higher | 1.06 [0.30,3.74] | 1.05 [0.30,3.66] | 1.08 [0.31,3.78] |  | 1.07 [0.31,3.71] | 1.06 [0.31,3.68] | 1.07 [0.31,3.71] | |
| **Household Wealth** |  |  |  |  |  |  |  |  |
| Poorest | 1.25 [0.55,2.87] | 1.24 [0.54,2.85] | 1.27 [0.58,2.78] |  | 1.26 [0.58,2.76] | 1.26 [0.57,2.76] | 1.26 [0.57,2.77] | |
| Poorer | 1.22 [0.68,2.21] | 1.22 [0.67,2.21] | 1.23 [0.69,2.21] |  | 1.23 [0.69,2.17] | 1.22 [0.69,2.17] | 1.23 [0.69,2.17] | |
| Middle | Reference | Reference | Reference |  | Reference | Reference | Reference | |
| Richer | 0.77 [0.31,1.92] | 0.78 [0.31,1.93] | 0.77 [0.31,1.92] |  | 0.77 [0.31,1.93] | 0.78 [0.31,1.95] | 0.77 [0.31,1.93] | |
| Richest | 0.67 [0.17,2.59] | 0.67 [0.17,2.59] | 0.67 [0.18,2.58] |  | 0.68 [0.18,2.59] | 0.68 [0.18,2.61] | 0.68 [0.18,2.60] | |
| **Place of Residence** |  |  |  |  |  |  |  |  |
| Urban | Reference | Reference | Reference |  | Reference | Reference | Reference | |
| Rural | 1.12 [0.86,1.46] | 1.10 [0.82,1.47] | 1.14 [0.87,1.49] |  | 1.13 [0.90,1.42] | 1.13 [0.90,1.41] | 1.13 [0.90,1.42] | |
| **Maternal BMI** |  |  |  |  |  |  |  |  |
| < 18.5 | 2.55*** [1.90,3.43] | 2.56*** [1.91,3.42] | 2.55*** [1.91,3.42] |  | 2.55*** [1.90,3.42] | 2.55*** [1.90,3.42] | 2.55*** [1.90,3.42] | |
| 18.5-24.9 | Reference | Reference | Reference |  | Reference | Reference | Reference | |
| 25.0-29.9 | 0.67 [0.42,1.08] | 0.67 [0.42,1.08] | 0.68 [0.42,1.08] |  | 0.67 [0.42,1.08] | 0.67 [0.42,1.07] | 0.67 [0.42,1.08] | |
| 30+ | 1.17 [0.78,1.76] | 1.17 [0.78,1.76] | 1.16 [0.77,1.76] |  | 1.16 [0.77,1.76] | 1.16 [0.77,1.76] | 1.16 [0.77,1.76] | |
| **Child’s Sex** |  |  |  |  |  |  |  |  |
| Male | Reference | Reference | Reference |  | Reference | Reference | Reference | |
| Female | 0.96 [0.62,1.48] | 0.96 [0.62,1.48] | 0.96 [0.62,1.49] |  | 0.96 [0.63,1.48] | 0.96 [0.63,1.47] | 0.96 [0.63,1.48] | |
| **Child’s Age** |  |  |  |  |  |  |  |  |
| 3 Years | Reference | Reference | Reference |  | Reference | Reference | Reference | |
| 4 Years | 0.93 [0.55,1.57] | 0.93 [0.55,1.58] | 0.93 [0.55,1.57] |  | 0.92 [0.55,1.55] | 0.92 [0.55,1.55] | 0.92 [0.55,1.55] | |
| **Anaemia Levels** |  |  |  |  |  |  |  |  |
| Severe | 2.88** [1.44,5.76] | 2.86** [1.42,5.78] | 2.89** [1.46,5.73] |  | 2.88** [1.46,5.68] | 2.87** [1.46,5.67] | 2.88** [1.46,5.68] | |
| Moderate | 1.30 [0.64,2.65] | 1.30 [0.64,2.66] | 1.31 [0.64,2.66] |  | 1.30 [0.64,2.65] | 1.30 [0.64,2.64] | 1.30 [0.64,2.65] | |
| Mild | 1.26 [0.66,2.40] | 1.25 [0.66,2.40] | 1.26 [0.67,2.40] |  | 1.26 [0.66,2.39] | 1.26 [0.66,2.38] | 1.26 [0.66,2.39] | |
| Not anaemic | Reference | Reference | Reference |  | Reference | Reference | Reference | |
| **Breastfeeding** |  |  |  |  |  |  |  |  |
| Ever breastfed, not currently | Reference | Reference | Reference |  | Reference | Reference | Reference | |
| Never breastfed | 1.71* [1.09,2.68] | 1.70* [1.09,2.65] | 1.72* [1.12,2.64] |  | 1.72* [1.10,2.67] | 1.71* [1.10,2.67] | 1.71* [1.10,2.67] | |
| Still breastfeeding | 1.54 [0.18,12.78] | 1.52 [0.18,12.73] | 1.54 [0.18,13.13] |  | 1.56 [0.20,12.33] | 1.57 [0.20,12.55] | 1.56 [0.20,12.43] | |

## S4: Association between childhood lifetime and recent experience of armed conflict and childhood underweight of children aged 36-59 years in Nigeria

|  | Lifetime experience of armed conflict and childhood underweight  Odd Ratios [95% Confidence Intervals] | | |  | Recent experience of armed conflict and childhood wasting  Odd Ratios [95% Confidence Intervals] | | | |
| --- | --- | --- | --- | --- | --- | --- | --- | --- |
| Frequency of attack | 2.33* [1.19,4.59] |  |  |  | 1.1 [0.94,1.29] |  |  | |
| Intensity of attack |  | 1.01** [1.00,1.03] |  |  |  | Reference |  | |
| Duration of attack |  |  | 1.02 [0.59,1.74] |  |  |  | 1.19*** [1.11,1.26] | |
| **Maternal Age Group** |  |  |  |  |  |  |  |  |
| 15-24 | Reference | Reference | Reference |  | Reference | Reference | Reference | |
| 25-34 | 0.99 [0.89,1.09] | 0.99 [0.89,1.09] | 0.99 [0.89,1.09] |  | 0.99 [0.89,1.10] | 0.99 [0.89,1.10] | 0.99 [0.89,1.10] | |
| 35+ | 0.9 [0.76,1.08] | 0.9 [0.76,1.08] | 0.91 [0.76,1.08] |  | 0.91 [0.76,1.08] | 0.91 [0.76,1.08] | 0.91 [0.76,1.08] | |
| **Maternal Education** |  |  |  |  |  |  |  |  |
| No education | Reference | Reference | Reference |  | Reference | Reference | Reference | |
| Primary | 0.8 [0.63,1.01] | 0.8 [0.63,1.01] | 0.79 [0.62,1.01] |  | 0.79 [0.62,1.00] | 0.79 [0.62,1.00] | 0.79 [0.62,1.01] | |
| Secondary | 0.69*** [0.55,0.85] | 0.69*** [0.55,0.85] | 0.68*** [0.55,0.85] |  | 0.68*** [0.55,0.85] | 0.68*** [0.55,0.85] | 0.68*** [0.55,0.85] | |
| Higher | 0.62*** [0.48,0.79] | 0.62*** [0.48,0.79] | 0.61*** [0.48,0.78] |  | 0.61*** [0.48,0.79] | 0.61*** [0.48,0.78] | 0.62*** [0.48,0.79] | |
| **Household Wealth** |  |  |  |  |  |  |  |  |
| Poorest | 1.25* [1.00,1.56] | 1.25* [1.00,1.55] | 1.24 [1.00,1.53] |  | 1.24 [1.00,1.55] | 1.23 [1.00,1.53] | 1.25* [1.01,1.54] | |
| Poorer | 1.19 [0.88,1.61] | 1.19 [0.88,1.60] | 1.18 [0.87,1.60] |  | 1.18 [0.88,1.60] | 1.18 [0.87,1.59] | 1.19 [0.88,1.60] | |
| Middle | Reference | Reference | Reference |  | Reference | Reference | Reference | |
| Richer | 0.70* [0.53,0.92] | 0.70* [0.53,0.92] | 0.70* [0.53,0.92] |  | 0.70** [0.53,0.92] | 0.70* [0.53,0.92] | 0.69** [0.53,0.92] | |
| Richest | 0.56* [0.32,0.98] | 0.56* [0.32,0.99] | 0.56* [0.32,0.97] |  | 0.55* [0.32,0.96] | 0.56* [0.32,0.97] | 0.55* [0.32,0.96] | |
| **Place of Residence** |  |  |  |  |  |  |  |  |
| Urban | Reference | Reference | Reference |  | Reference | Reference | Reference | |
| Rural | 1.15 [0.88,1.49] | 1.15 [0.89,1.49] | 1.13 [0.86,1.46] |  | 1.13 [0.88,1.47] | 1.12 [0.87,1.45] | 1.14 [0.89,1.46] | |
| **Maternal BMI** |  |  |  |  |  |  |  |  |
| < 18.5 | 1.36 [0.93,2.00] | 1.36 [0.93,2.00] | 1.36 [0.93,2.00] |  | 1.36 [0.93,2.00] | 1.36 [0.93,2.00] | 1.36 [0.93,2.00] | |
| 18.5-24.9 | Reference | Reference | Reference |  | Reference | Reference | Reference | |
| 25.0-29.9 | 0.75 [0.56,1.00] | 0.75 [0.56,1.00] | 0.74* [0.56,1.00] |  | 0.75 [0.56,1.00] | 0.74* [0.56,1.00] | 0.75 [0.56,1.00] | |
| 30+ | 0.47*** [0.31,0.72] | 0.47*** [0.31,0.72] | 0.47*** [0.31,0.73] |  | 0.47*** [0.31,0.73] | 0.47*** [0.31,0.74] | 0.47*** [0.30,0.74] | |
| **Child’s Sex** |  |  |  |  |  |  |  |  |
| Male | Reference | Reference | Reference |  | Reference | Reference | Reference | |
| Female | 1.03 [0.97,1.10] | 1.03 [0.97,1.10] | 1.03 [0.97,1.09] |  | 1.03 [0.97,1.10] | 1.03 [0.97,1.10] | 1.03 [0.97,1.10] | |
| **Child’s Age** |  |  |  |  |  |  |  |  |
| 3 Years | Reference | Reference | Reference |  | Reference | Reference | Reference | |
| 4 Years | 0.88*** [0.81,0.95] | 0.88*** [0.81,0.94] | 0.88** [0.82,0.95] |  | 0.88** [0.82,0.95] | 0.88** [0.82,0.95] | 0.88** [0.82,0.95] | |
| **Anaemia Levels** |  |  |  |  |  |  |  |  |
| Severe | 3.52*** [2.66,4.66] | 3.52*** [2.66,4.67] | 3.51*** [2.66,4.64] |  | 3.51*** [2.66,4.64] | 3.51*** [2.65,4.64] | 3.52*** [2.67,4.65] | |
| Moderate | 1.98*** [1.63,2.39] | 1.98*** [1.63,2.39] | 1.97*** [1.62,2.39] |  | 1.97*** [1.63,2.39] | 1.97*** [1.62,2.39] | 1.98*** [1.63,2.39] | |
| Mild | 1.54** [1.15,2.06] | 1.54** [1.15,2.06] | 1.54** [1.15,2.05] |  | 1.54** [1.15,2.06] | 1.54** [1.15,2.05] | 1.54** [1.16,2.06] | |
| Not anaemic | Reference | Reference | Reference |  | Reference | Reference | Reference | |
| **Breastfeeding** |  |  |  |  |  |  |  |  |
| Ever breastfed, not currently | Reference | Reference | Reference |  | Reference | Reference | Reference | |
| Never breastfed | 1.80*** [1.35,2.39] | 1.80*** [1.35,2.39] | 1.79*** [1.34,2.39] |  | 1.79*** [1.35,2.39] | 1.79*** [1.34,2.39] | 1.80*** [1.35,2.40] | |
| Still breastfeeding | 1.36 [0.55,3.39] | 1.37 [0.55,3.39] | 1.36 [0.54,3.40] |  | 1.35 [0.54,3.38] | 1.36 [0.54,3.42] | 1.35 [0.54,3.35] | |
